# Give Me a Sign: Using Data Gloves for Static Hand-Shape Recognition

**DOI:** 10.3390/s23249847

**Published:** 2023-12-15

**Authors:** Philipp Achenbach, Sebastian Laux, Dennis Purdack, Philipp Niklas Müller, Stefan Göbel

**Affiliations:** Serious Games Group, Technical University of Darmstadt, 64289 Darmstadt, Germanydennis.purdack@hoems.hessen.de (D.P.); stefan_peter.goebel@tu-darmstadt.de (S.G.)

**Keywords:** machine learning, classification, support vector machines, random forest classifier, logistic regression, voting meta-classifier, outlier detection, feature selection, data augmentation, hand-shape recognition, sign language, virtual reality

## Abstract

Human-to-human communication via the computer is mainly carried out using a keyboard or microphone. In the field of virtual reality (VR), where the most immersive experience possible is desired, the use of a keyboard contradicts this goal, while the use of a microphone is not always desirable (e.g., silent commands during task-force training) or simply not possible (e.g., if the user has hearing loss). Data gloves help to increase immersion within VR, as they correspond to our natural interaction. At the same time, they offer the possibility of accurately capturing hand shapes, such as those used in non-verbal communication (e.g., thumbs up, okay gesture, …) and in sign language. In this paper, we present a hand-shape recognition system using *Manus Prime X* data gloves, including data acquisition, data preprocessing, and data classification to enable nonverbal communication within VR. We investigate the impact on accuracy and classification time of using an *outlier detection* and a *feature selection* approach in our data preprocessing. To obtain a more generalized approach, we also studied the impact of artificial *data augmentation*, i.e., we created new artificial data from the recorded and filtered data to augment the training data set. With our approach, 56 different hand shapes could be distinguished with an accuracy of up to 93.28%. With a reduced number of 27 hand shapes, an accuracy of up to 95.55% could be achieved. The voting meta-classifier (VL2) proved to be the most accurate, albeit slowest, classifier. A good alternative is random forest (RF), which was even able to achieve better accuracy values in a few cases and was generally somewhat faster. *outlier detection* was proven to be an effective approach, especially in improving the classification time. Overall, we have shown that our hand-shape recognition system using data gloves is suitable for communication within VR.

## 1. Introduction

In everyday communication, non-verbal language plays an important role alongside verbal language, for example, through the use of hand gestures. They help us to express feelings and thoughts, to give context to spoken language (e.g., by pointing to something while speaking), or even to replace spoken language completely (e.g., by using the *thumbs up* gesture to signal to another person that everything is okay).

With technological progress, the desire to transfer this natural way of interpersonal interaction to computers is increasing. Thus, machines could be controlled directly using gestures: instead of the user learning to control the machines, they would use natural and instinctive means of communication, and the machine would learn to understand them.

In addition, even within a computer-generated environment, such as VR, interpersonal communication could be accomplished through the use of gestures. This would allow simple hand gestures, such as the aforementioned *thumbs up* gesture, to be used in the context of operational force training. Significantly more complex issues could also be presented in the context of sign language, either because this is given by the application (e.g., sign learning software ) or because this is the user’s primary form of communication, e.g., for deaf and hard of hearing people. This is an aspect that is becoming increasingly important because, according to the World Health Organization [1], there are approximately 430 million people worldwide with some degree of hearing loss, and the trend is increasing. Where hearing people within VR communicate predominantly with microphones in their spoken language, the deaf and hard of hearing have to express themselves non-verbally, for example, via a chat function. In addition, they cannot hear when other users communicate via the microphone. There are speech-to-text solutions that can display the spoken word as text, but an approach that can also convert signs into text or speech would still have to be developed for bidirectional communication.

Developing a system that can recognize and translate signs requires first determining how signs are structured. Linguist *William C. Stokoe* [2,3] was one of the first to break down signs into their characteristic components. According to him, a sign consists essentially of the parameters of the hand shape, the orientation of the hand, the movement of the hand, and the location of execution of the sign. Other non-manual parameters such as facial expression are also conceivable, but the most important parameter is the hand shape [4]. This is also evident when looking at the American Sign Language (ASL) finger alphabet: There are 26 signs with a total of 21 different hand shapes, which are all performed with the dominant hand. Two pairs of signs have the same hand shapes, but differ by having a movement of the hand (*I*⇔*J* and *1*⇔*Z*). Three other pairs of signs have the same hand shapes, but differ in the orientation of the hand (*K*⇔*P*, *G*⇔*Q*, and *H*⇔*U*).

To determine the hand shapes of an entire vocabulary, a suitable data set is needed. *ASL-Lex* is a public sign lexicon for ASL [5,6]. It contains videos and information on 2723 signs. One component of this information is the so-called *Phonological Coding System*, which is based on the *Prosodic Model of Sign Language* by Brentari [7]. It describes signs based on their characteristic features, similar to the aforementioned notation system of Stokoe [3], only with significantly more parameters. To the best of our knowledge, there is no other publicly accessible database of this size that displays gestures in parametric form.

To recognize hand shapes reasonably, it also needs the appropriate hardware. There are different approaches, which can be distinguished in particular into video-based and (other) sensor-based approaches. In VR, data gloves are often used as an alternative to traditional controllers because they can capture hand shapes and movements even in complex motion sequences and are independent of occlusions [8].

The main application of data gloves is hand-gesture recognition, especially for static gestures. Between 2015 and 2022, more than 100 papers were published in English on this topic in reputable sources, like the Institute of Electrical and Electronics Engineers (IEEE) or the Association for Computing Machinery (ACM), according to the Web of Science (WoS). More than 70% of these examine static gestures [9].

Even though these papers all pursue the topic of hand gesture recognition with data gloves, they differ in some points: (*i*) used classification methods, (*ii*) number of participants, (*iii*) number of samples, (*iv*) number of hand gestures, (*v*) type of hand gestures. The type of hand gestures is defined by the used features. The more features are present, the more information is available for the classifier to successfully recognize the hand gesture. Therefore, many of these papers ([10,11,12]) not only use hand-shape information for classification, but also add hand orientation as an additional feature.

A distinction is also made between static and dynamic gestures: Static gestures possess spatial information, like the already-mentioned hand shape, the orientation of the hand, or the location of the hand where the gesture is performed. Dynamic gestures additionally possess temporal information, such as the movement of the hand [12], the rotation of the ulnar, or a change in finger pose (e.g., closed fingers that are spread) [5]. Therefore, some of the papers use dynamic gestures instead of static ones [10,12,13,14].

### Goal and Methodology

**Figure 1:**
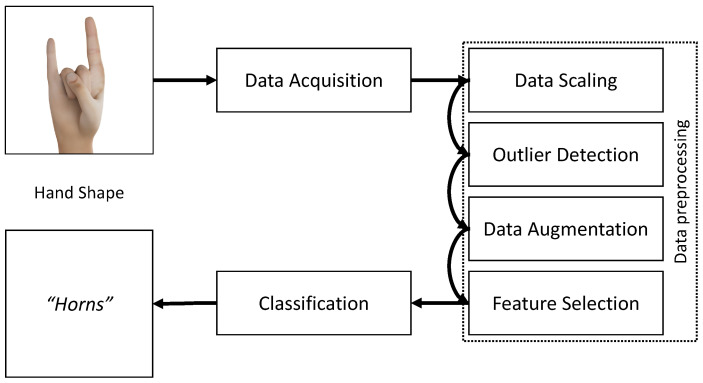
Our classification pipeline.

In this study, we focus on the recognition of static hand shapes with data gloves. We investigate whether commercially available data gloves are suitable for recognizing hand shapes of sign language in the use of VR. For this purpose, we designed a classification pipeline to reliably detect static hand shapes using a generalizable approach that can be used for other static data. The individual steps of data preprocessing will be examined with respect to their performance (accuracy and time for classification) and a recommendation is made as to which steps should be used for which use case. The classification pipeline can be seen in Figure 1.

First, we acquire the hand shape with a *Manus Prime X* data glove. To ensure high-quality data, an *outlier detection* method called *Density-Based Spatial Clustering of Applications with Noise (DBSCAN)* is applied to the training data. The data are further augmented using a proprietary method and thus artificially duplicated with the goal of counteracting overfitting of the classification. To reduce the amount of training data and increase the speed of training and classification, we apply *feature selection* in the form of a *genetic algorithm (GA)*.

For evaluation, we chose two different data sets: 27 hand shapes from the ASL finger alphabet (letters and numbers) and 56 hand shapes from a 2700+ word lexicon of ASL. On the one hand, this covers a variety of different hand shapes and, at the same time, serves to be able to create a basis for a sign language application within VR.

Our pipeline is generic and can be applied to any type of static data as long as they are in the correct data format. However, it is recommended to adjust the various parameters of the pipeline, such as the hyperparameters of the classifiers, to the new data.

## 2. Data Acquisition

To reliably recognize hand shapes, they must be recorded in a suitable form. The recordings can then be used to train machine learning (ML) classifiers. Attention must be paid to the choice of suitable hardware and the selection of features to be captured.

**Figure 2:**
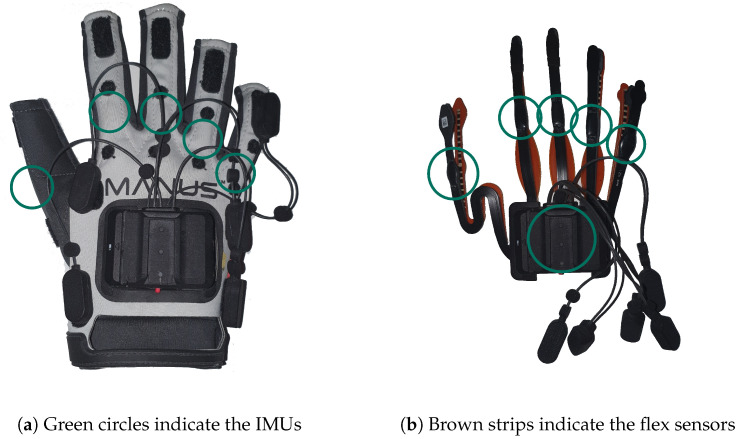
Manus Prime X data glove and bare sensors of the glove [15].

The data gloves that were used during our experiment are the *Manus Prime X Haptic* (https://www.manus-meta.com/products/prime-x-haptic (last visited on 21 October 2023)) and are specifically designed for use within VR. The gloves can be seen in Figure 2.

**Figure 3:**
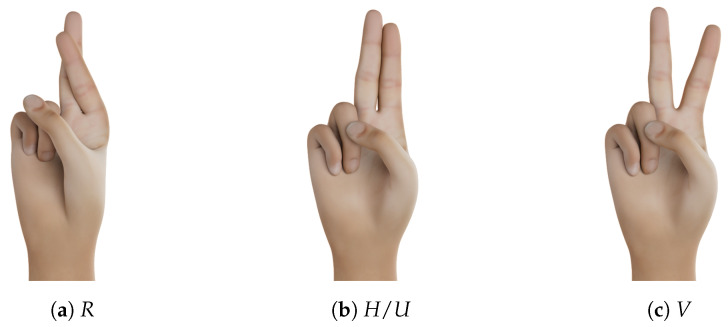
Hand shapes *R*, *H* (identical to *U*, only different in orientation,) and *V* differ only in spread.

A 9-degrees of freedom (DoF) inertial measurement unit (IMU) and a 2D flex sensor are attached to each finger to obtain reliable values about the flexion/stretch of the fingers and the spread between each finger. The latter information is not available in some data gloves, but is indispensable for distinguishing individual hand shapes such as *R*, *U*, and *V* (see Figure 3) [9]. A 6-DoF IMU is attached to the back of the hand to obtain its orientation. The accuracy of each finger measurement is ±2.5 degrees.

The acquired sensor values are internally fused and preprocessed with the *Manus Core C++ SDK* (https://documentation.manus-meta.com/v1.9.0/cpp-sdk/ (last visited on 21 October 2023)). The preprocessed data are transferred to the computer via Bluetooth. According to the manufacturer, the latency is less than 5 ms, and the glove’s sensor sampling rate is 90 Hz. Table 1 shows all spread and stretch values given by the SDK that we use as features to represent each static gesture.

To obtain the best-quality sensor data, the gloves must be calibrated. This is achieved by performing three simple gestures via the SDK. This also compensates for deviations that may occur due to differently sized user hands. The calibration is stored in the glove so that it is immediately ready for use for the next session.

## 3. Data Preprocessing

The main idea of *data preprocessing* is to highlight important information in the available data while also removing some of the redundant or misleading data that may be present [10].

The first step of *data preprocessing* is often to scale all data to a predetermined interval. The intervals [0,1] or [−1,1] are often used. Alternatively, statistical properties of the training data can be used for scaling. In this study, we use sklearn’s *StandardScaler* (https://scikit-learn.org/stable/modules/generated/sklearn.preprocessing.StandardScaler.html (last visited 27 September 2023)). It calculates the average value of each feature *i*, subtracts it from each data point, and divides it by the standard deviation.

$$Z_{i}=\frac{X_{i}-\mu_{i}}{\sigma_{i}}$$

In this way, all features follow a normal distribution with zero mean and unit variance. We chose this scaling procedure because it has been shown that some models, such as support vector machine (SVM) or certain linear models, may perform worse when the data are not scaled and centered around zero [16,17].

Other than scaling the data, we implemented several *outlier detection* and *feature selection* methods that sort out misleading data samples or unimportant features. We also experimented with various *data augmentation* techniques to artificially enrich our data set with the goal of improving the generalizability of our approach. The best methods in each category are presented in the following.

### 3.1. Outlier Detection

Outliers are samples that differ greatly from the other recorded samples. Outliers can occur during data acquisition, for example, due to sensor drift or because a user performs a gesture incorrectly. Such outliers can negatively impact the performance of ML classifiers, as it is often best for these models to be able to generalize and not overfit the data [18]. *Outlier detection* therefore aims to find and remove all outliers within the given data.

Many algorithms used to achieve this goal are similar to clustering algorithms in that samples are also combined to form clusters. Points that do not belong to any cluster are then identified as outliers. In this study, we used the *DBSCAN algorithm*. DBSCAN starts at a random data point and searches for other samples within a predefined distance ε. If the number of samples within this distance is greater than the *minPoints* parameter, the original point is marked as a *core point*. This step is repeated for all data points. Afterwards, a random core point is selected and the point itself and all neighboring points within ε are added to a cluster. When all core points have been assigned to a cluster, the algorithm terminates. All samples that are not part of any cluster are considered outliers.

**Figure 4:**
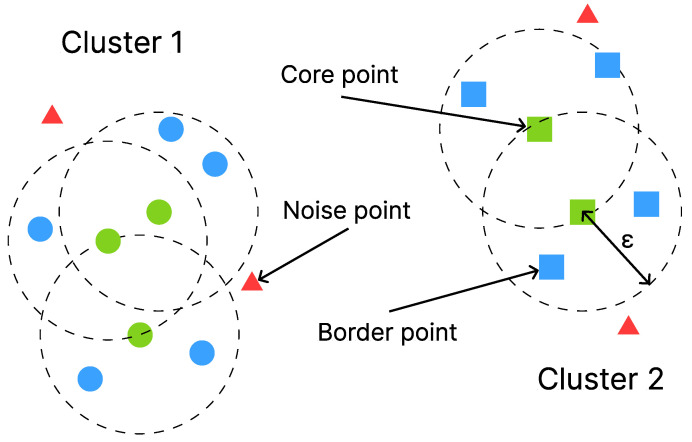
DBSCAN assigning points into core, border, and noise points (https://www.kdnuggets.com/2020/04/dbscan-clustering-algorithm-machine-learning.html (last visited 21 October 2023)).

The algorithm can be controlled with the ε and *minPoints* parameters. Figure 4 shows how DBSCAN assigns multiple points into two clusters. *minPoints* is set to four in this example (https://www.kdnuggets.com/2020/04/dbscan-clustering-algorithm-machine-learning.html (last visited 21 October 2023)).

*Outlier detection* is rarely used in other works on gesture recognition. Most often, outliers are removed from the test set. This is usually carried out when outliers and incorrect predictions in the application phase can have serious consequences, such as in the medical field. In these scenarios, it is often more favorable to detect outliers and output a warning alongside the models’ prediction. Related works that operate in this way are, for example, by Zhang et al. [19] or by Palipana et al. [20].

In this study, we focused on detecting outliers only in the training data, since our application phase is not as critical at this time and it can be more easily compared to most other gesture recognition work. Test data should also, in our opinion, represent a possible real-world scenario; this includes biases in sensor values or erroneous user executions.

Once the *outlier detection* has been performed, the remaining data are scaled again according to the principle described above.

### 3.2. Data Augmentation

In order to use ML models for reliable hand-shape classification, a sufficient amount of high-quality training data must be available. In particular, in the application area of gesture recognition with the use of wearable sensors as a data source, the acquisition of large amounts of data for learning poses is one of the main challenges due to the cumbersome acquisition process. This is because the data must be physically gathered from individuals equipped with wearable sensors and then carefully labeled afterwards (see Section 2), which takes time and effort and usually yields inadequate quantities, especially for *deep learning* approaches [21]. In addition, further challenges may arise, for example, at the time of the *COVID-19 pandemic*, which required even stricter hygiene standards and therefore may increase the cost of physical data acquisition with wearable sensors. Thus, building a rich and diverse data set may become even more laborious. This potential scarcity of training data can then lead to poor generalization capabilities of the model.

One way to deal with these problems is the use of *data augmentation* to artificially enrich the training data set. This is usually achieved by applying transformations to the existing data to create new, synthetic data samples. *Data augmentation* can therefore be employed as a preprocessing step in order to ultimately reduce overfitting and enhance the robustness and generalizability of the ML models used [22].

However, the applicability of different *data augmentation* methods depends on the type of data available and corresponding sensor technology, and therefore must be evaluated for the specific task at hand. Depending on these factors, and additionally on the ML classifiers used, the effectiveness of *data augmentation* may vary.

As introduced in Section 2, static spread and stretch values for each joint are used in this study as features. However, the available literature on *data augmentation* for wearable sensors is mainly concerned with dynamic data consisting of a gesture performed within a certain time interval. For example, Um et al. [22] conducted one of the most comprehensive evaluations of *data augmentation* techniques for wearable sensor data used in dynamic approaches. These methods leverage variations in orientation or timing and are therefore not applicable in this setting, since the available data do not capture positional or dynamic properties. In contrast, the literature on *data augmentation* for static hand-shape recognition is rather scarce and mostly not the subject of studies. However, in this study, we have adapted a *data augmentation* approach presented by Liu and Ostadabbas [23] so that it is applicable to the available data and can be used in our setting to reduce overfitting and improve the generalizability of the models by introducing more variety to the way hand shapes are performed. Below, we present the *data augmentation* approach we use to generate artificial data samples, i.e., hand shapes.

#### Methodology

We have adopted and slightly adapted a *data augmentation* approach presented by Liu and Ostadabbas [23], where joint angle constraints are used to define range boundaries for each joint. Using these range boundaries, new poses can be generated by randomly sampling within the defined limits for each joint. This ensures that the newly generated data samples are valid, since the boundaries can be set appropriately.

**Figure 5:**
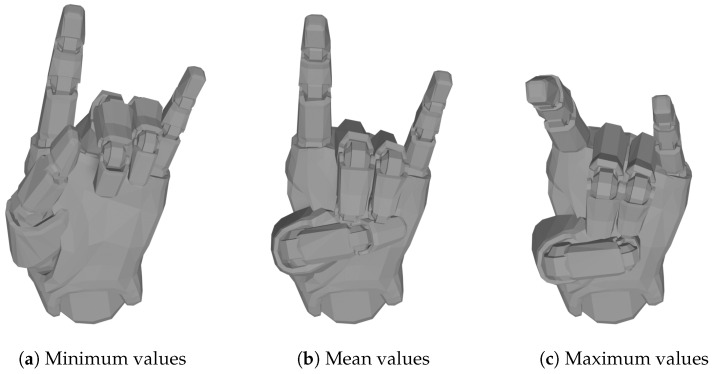
Minimum, mean, and maximum values of hand shape *Horns*.

We modified this approach to generate new data samples for each specific hand shape (i.e., label). Therefore, these range boundaries must be chosen differently for each hand shape and define the amount of maximum and minimum joint bending that is still considered to be the respective hand shape. This would be required for each hand shape in our data set. In this study, we define the limits by first calculating the respective minimum and maximum joint values for each hand shape from the available data. As an example, Figure 5a,c show the minimum and maximum hand shape for the *Horns* label, which is illustrated in Table A1 in Appendix A (we used the 3D hand model provided by the *Manus Core* Plugins and visualized it using *Blender* [24]). Compared to the mean hand shape in Figure 5b, it becomes apparent that there may be slight variations in the way a hand shape is performed by individuals due to anatomical differences, which may lead to larger disparities in some feature values depending on the hand shape. In addition, inaccuracies in the glove data sensors also seem to play a role, because although the hand shapes were recorded under supervision and performed again in case of errors, there are still sometimes large differences in the data. These two factors can lead to large differences in some joint values, especially in the joints of the thumb.

In order to improve generalizability to yet-unseen data samples, we further add (subtract) half the standard deviation of each feature $f_{i}$ to (from) the calculated maximum (minimum) values for each hand shape (i.e., label *l*). Since we have a normal distribution with unit variance (σ=1) due to standardization, the calculation simplifies as follows:

$${\tilde{f}}_{i}^{min}=f_{i}^{min}-\frac{\sigma }{2}=f_{i}^{min}-\frac{1}{2}$$

$${\tilde{f}}_{i}^{max}=f_{i}^{max}+\frac{\sigma }{2}=f_{i}^{max}+\frac{1}{2}$$

Concretely, this results in minimum values with feature vector

$$F_{l}^{min}={\left({\tilde{f}}_{0}^{min},\;{\tilde{f}}_{1}^{min},\;{\tilde{f}}_{2}^{min},\;\ldots ,\;{\tilde{f}}_{n-1}^{min}\right)}^{T}$$

and maximum values with feature vector

$$F_{l}^{max}={\left({\tilde{f}}_{0}^{max},\;{\tilde{f}}_{1}^{max},\;{\tilde{f}}_{2}^{max},\;\ldots ,\;{\tilde{f}}_{n-1}^{max}\right)}^{T}$$

for each label *l*. Here, n=20 is the number of features, as shown in Table 1. Using these limits, a new data sample

$${\hat{F}}_{l}={\left({\hat{f}}_{0},\;{\hat{f}}_{1},\;{\hat{f}}_{2},\;\ldots ,\;{\hat{f}}_{n-1}\right)}^{T}$$

can then be generated for a specific label *l* by sampling new feature values ${\hat{f}}_{i}$ from a uniform distribution $U(f_{i}^{min},f_{i}^{max})$ where i∈[0,n−1].

Even after applying *data augmentation*, the entire data set, including the augmented data, is scaled again, as described at the beginning of the chapter.

### 3.3. Feature Selection

**Figure 6:**
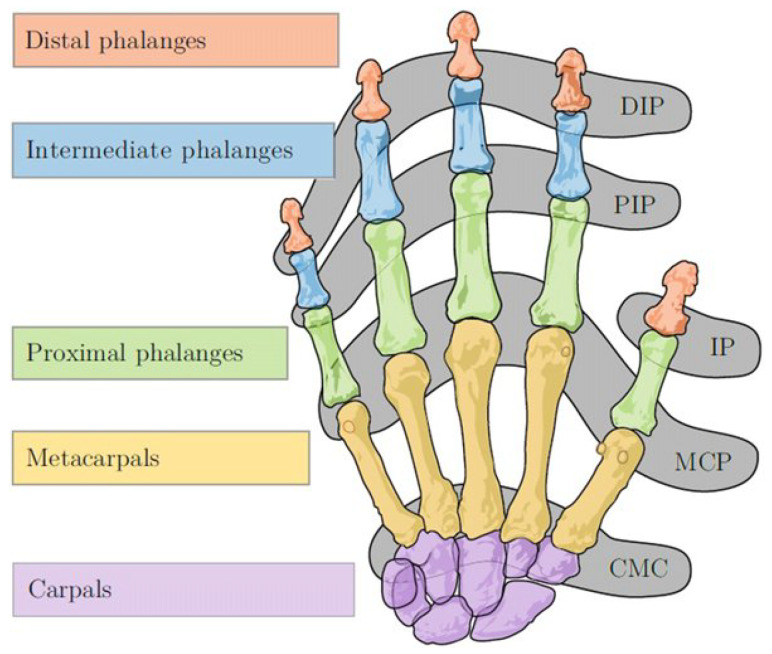
Joints and bones of the human hand [25].

Each feature of a data sample holds a certain amount of information about the performed gesture. Some features may be more important than others. For example, the *distal interphalangeal (DIP)* and *proximal interphalangeal (PIP)* joints, shown in Figure 6, are interdependent in most of the gestures that were investigated here [9]. Consequently, only one of these features holds significant information about the performed gesture. This is in contrast to any of the thumb features, as the exact position of the thumb plays an important role in many of gestures that were examined in this study. Thus, in general, many of the *DIP* and *PIP* features hold very little information about the performed gesture, while other features, such as that of the thumb, are more important for the classification.

*Feature selection* takes advantage of this and tries to keep the most important features, while also removing features that hold very little information. That way, fewer features are used to represent a single gesture, meaning the data take up less space and the ML models can focus on the most important data [26].

In this study, we used a genetic algorithm (GA) for *feature selection*. The algorithm is loosely based on the theory of evolution and consists of an initialization phase and four repeating phases after that (a step-by-step instruction can be found at https://neuraldesigner.com/blog/genetic_algorithms_for_feature_selection#GeneticAlgorithms (last visited 21 October 2023)): (*i*) At first, multiple bitstrings are randomly created (initialization). Each bit corresponds to a single feature that is either kept (1) or discarded (0). For each of these bitstrings, one ML model is created and trained with the corresponding features. (*ii*) Afterwards, some of these models are selected for the next phase of the algorithm. Models with high accuracy often have a higher chance to be selected by the algorithm. (*iii*) The remaining bitstrings are combined to form new combinations of features. (*iv*) These may randomly flip single bits (= *mutation*). The resulting bitstrings are used to train new models. The whole process is repeated until either a predefined number of iterations is reached or there has not been a significant accuracy improvement for multiple iterations [27].

The algorithm has also been used in related studies, such as that of Li et al. [28], to reduce the training error alongside the number of epochs of a neural network, when classifying ten gestures. Without GA, a training error of about 0.00566 was reached after 5000 epochs. Using GA, the error was reduced to about 0.00042. Using a handcrafted modification of the GA reduced the error to about 0.00010 after just 608 epochs.

## 4. Machine Learning Classification

Building on the results of Achenbach et al. [11], we chose the classifiers support vector machine (SVM), random forest (RF), and Logistic regression (LR) for our investigations, as they were able to achieve the highest accuracy values in a similar experiment. We added a voting meta-classifier (VL2) to combine the advantages of all these classifiers. In comparison, we are now using different hardware and a larger number of gestures: Achenbach et al. [11] examined five gestures with fifteen features of hand shape and twenty-five gestures with the same fifteen features of hand shape plus four additional features for hand orientation. In this study, we examine 27 and 56 gestures with 20 features of hand shape.

In the following, we briefly present the rough working of each classifier and explain the conditions under which we used them.

### 4.1. Support Vector Machine

SVMs are used to split data into two classes. This is achieved by mapping the data into a vector space and looking for a linear hyperplane that separates the data according to the max-margin paradigm. This generally results in less overfitting and more robustness when classifying unseen data. Projecting the data into a higher-dimensional vector space, finding a linear hyperplane there, and projecting the data and hyperplane back into the original vector space can transform the hyperplane from a linear function to one of a higher complexity. This procedure is used to classify data that are not linearly separable. In practice, the so-called *kernel trick* is often used instead of transforming the entire vector space to save computing time [29].

Following this procedure, a single SVM can differentiate between two classes. However, most classification problems contain more than just two output classes. In multi-class classification problems, more than one SVM has to be used to separate the data. There are two commonly used methods to train these SVMs. In the *one-versus-one (OvO)* approach, one SVM is created for every pair of classes. The final decision is often made by performing a majority vote over all SVMs. In the other method, *one-versus-all (OvA)*, a single SVM is trained for each class and is used to distinguish between that class and all the other classes. The final output is usually provided by the SVM with the highest confidence score [30]. In this study, we used the OvO approach to classify all of our data.

### 4.2. Random Forest

An RF uses the results of multiple decision trees (DTs) to calculate its own prediction. A single DT within an RF often performs worse than a full-fledged DT. This is because a single tree inside an RF is usually trained on a small subset of the data and its features. The subset is generated by sub-sampling the original training data with replacement [29]. It is important to have different subsets for most of the trees. The idea is to train a large number of diverse DTs. Each one may heavily focus on one part of the training data, while neglecting other parts, thus being worse than a DT trained with all the available data [31]. However, their results are then combined, often by a majority vote. Together, they usually perform better than a single DT, while overfitting less and thus generalizing better.

It has been shown that RFs do not overfit by increasing the number of trees [31]. Hundreds or thousands of trees are often trained when using RFs. One advantage of training so many classifiers is that they can also be used to analyze the data. For example, adding noise to a single feature and observing the change in accuracy of all DTs can be an indication of the importance of that specific feature [29]. The large number of classifiers ensures that a higher error rate is actually caused by the random noise added to the feature and not by a specific characteristic of a single classifier.

### 4.3. Logistic Regression

In the most basic case of LR, the model has to distinguish between two output classes. In this case, LR calculates the probability of a sample belonging to one of the two output classes. If the calculated value exceeds 50%, the sample is assigned to that class. Otherwise, the other output class is chosen. Probabilities close to either 0% or 100% are often desirable because the model is sure about assigning the corresponding sample to one of the two classes in these cases. Probabilities close to 50% are very susceptible to small amounts of noise. The probability is often calculated using the *logit* function [32],

$$\mathrm{logit}(p)=ln\left(\frac{p}{1-p}\right)=\beta_{0}+\sum \beta_{i}*X_{i},$$

where $X_{i}$ represent the features of the data. The $\beta_{i}$ are weights that must be calculated when fitting the model. The resulting function usually follows the shape of a sigmoid. In general, a steeper slope leads to better predictions, as there are fewer inputs with probabilities close to 50% this way.

When classifying more than two classes, LR uses a similar strategy to that presented in Section 4.1. Because of the higher training times when using large amounts of data, most of the time the OvA approach is used for LR instead of OvO.

### 4.4. Voting Meta-Classifier

A meta-classifier is a model that does not operate on the input data alone. It uses other models to improve its own predictions. This way, the entire system becomes more resistant to failures of individual models or sensors, as well as noise in the data [33]. Such models are often organized in layers. The voting classifier used in this study consists of two layers. The three classifiers presented in this chapter form the first layer. These models use the input data to predict the output class. The second layer is the voting classifier itself. It combines the predicted probabilities of the models in the previous layer to produce its own output based on the argmax of their sums. A weighted average, where the weights are based on the grid search results of the classifiers in the first layer, produced the best results.

***Remark**: Deep-Learning Algorithms*

In the specified preliminary study [11], the *deep-learning* algorithm feedforward neural networks (FNN) were tested with comparable hardware and data. A *Senso Glove: DK2* data glove was used, which also enables IMU-based tracking of the hands and fingers, but does not use flex sensors. The recorded data were hand shapes from the well-known game rock–paper–scissors (RPS), all of which are very similar and sometimes even identical to the hand shapes recorded in this paper. In the larger data set with 25 different hand shapes, the orientation of the hand was taken into account and 19 features were used. The smaller data set with five different hand shapes, all of which are identical to the hand shapes from this paper, used 15 features without the orientation of the hand.

FNN did not perform better than traditional classifiers such as SVM, RF, or LR with this type of hardware and data, but required significantly more effort to design and optimize. The classifiers just mentioned achieved higher accuracies, even if FNN was sometimes able to classify faster. We assume that the reason for this is that our classification problem (static gestures with only 20 features) is relatively straightforward. The design of neural networks would be considerably more complex and would significantly increase the length of the paper. We therefore decided not to use *deep-learning* algorithms in this paper and to focus on the classifiers presented so far.

## 5. Experiment

In an experiment [15], different hand shapes used in the *ASL-Lex* lexical database (https://asl-lex.org (last visited 27 September 2023)) and ASL manual alphabet (including digits) were recorded with a *Manus Prime X* data glove. We examined two different data sets in this paper:

**ASL manual alphabet**  consists of 26 different hand gestures with 21 different hand shapes. To represent the digits 0–9 as well, six more hand shapes were added. This leads us to 27 hand shapes with which fingerspelling is possible, i.e., the possibility to spell names and numbers.
**ASL-Lex**  uses 58 different hand shapes for the dominant hand. For reasons we cannot explain, the hand shapes *Flat H* and *Flat N* are displayed identically (comparison of https://aslcdi.website/images/handshape_images/flat_h.png and https://aslcdi.website/images/handshape_images/flat_n.png (last visited 16 November 2023) [5]) in ASL-Lex and cannot be distinguished. We therefore combine them and refer to them as *Flat N*. As already mentioned, the letters of the finger alphabet *P* and *K* also share the same hand shape (comparison of https://aslcdi.website/images/handshape_images/k.png and https://aslcdi.website/images/handshape_images/p.png (last visited 16 November 2023) [5]) and differ only in their orientation. We therefore have only considered *K*. Thus, we have a total of 56 unique hand shapes, which (together with other details such as movement or orientation of the hand) allow a vocabulary of more than 2700 characters.

All hand shapes from the ASL manual alphabet are found in the set of hand shapes of *ASL-Lex*, with the exception of the hand shape *M* and *N*. Therefore, we have a total set of 58 hand shapes, which are shown in Table A1.

Since the focus of this study is on hand-shape recognition, all recorded hand gestures are static, differ only by hand shape, and are independent of hand orientation. Therefore, all stretch and spread values from Table 1 were used as features. The quaternions of the individual fingers were not considered due to their dependence on orientation.

### 5.1. Data Acquisition

For data acquisition, a total of 20 participants took part in the experiment [15]. The experiment was conducted as follows:

To allow for better hand mobility, the vibration motors on the gloves were removed. Prior to each experiment, the gloves were recalibrated using the associated software of *Manus Core SDK* to clean up any possible drift in the IMU sensors and to ensure that different hand sizes of the participants did not affect the results.

After calibration, each participant sat at a table and was shown a picture of the hand movement to be performed. Pressing the *Enter* key started the recording. The participant then had three seconds to perform the hand gesture and then held it for an additional two seconds. In a later segmentation, the static hand shape was then extracted as one keyframe from the middle of this second section.

After recording, participants were asked to return their hands to the starting position and place them on the table. This process was repeated three times for each hand gesture. Throughout the experiment, participants were under observation to ensure that the hand gestures were performed correctly. Incorrect recordings were repeated at the end of the experiment.

Thus, for each of the 58 hand gestures we used, three repetitions were recorded for 20 participants, yielding a total of 3480 samples.

### 5.2. Hyperparameters

To find suitable hyperparameters for our hand-shape recognition system, we first performed a pre-grid search with ten-fold cross-validation over all recorded samples for both data sets and each combination of our data preprocessing methods. The hyperparameters were searched in the same areas that Achenbach et al. [11] already used. In this way, we were able to determine 16 different configurations of hyperparameters. From these, we have now defined a smaller, but more precise range, which can be viewed in Table 2. This range was used in each run of our following experiments with a five-fold cross-validation grid search.

To save computational resources, we performed the grid search based on the successive halving algorithm and used sklearn’s *HalvingGridSearchCV* (https://scikit-learn.org/stable/modules/generated/sklearn.model_selection.HalvingGridSearchCV.html (last visited 7 November 2023)). This algorithm allocates resources dynamically and favors the most promising hyperparameter configuration. Starting with an equal distribution of resources, the grid search, therefore, iteratively excludes hyperparameter combinations that are considered to be the least effective. Overall, this leads to considerable time savings in the search for the best hyperparameter configuration.

### 5.3. Hardware

An *Apple MacBook Pro* (https://support.apple.com/kb/SP858 (last visited 15 November 2023)) (16${}^{\prime \prime }$, 2021) with an *Apple M1 Max processor* (10-core CPU with 8 performance cores and 2 efficiency cores, 32-core GPU, 16-core neural engine, and 400 GB/s memory bandwidth) and 32 GB RAM was used to compute the results presented here. The Python library scikit-learn (https://scikit-learn.org/stable/ (last visited 28 September 2023)) (version 1.2.1) and Python (version 3.9.6) were used.

## 6. Results

The four classifiers were evaluated using a *leave-one-out* cross-validation, i.e., the training and test data were separated such that one participant’s data were used as test data and all other data were used as training data. In this way, all possible combinations were iterated, i.e., 20 repetitions for n=20 participants. To compare the performance of the classifiers, the accuracy and time for classification were stored and evaluated. The mean and standard deviation were calculated from the data thus obtained. Since we had an equal class distribution and prioritized each class equally, we omitted other measures such as the F-score.

Table 3 and Table 4 show the accuracy values of all classifiers with respect to the data preprocessing methods used. The best results for each classifier and data preprocessing configuration are marked in green; the worst results are marked in red.

**Figure A1:**
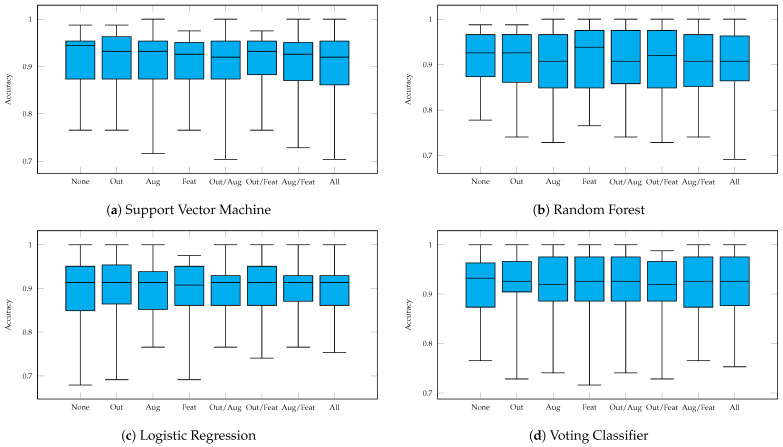
Accuracy values in leave-one-out cross-validation for classifiers with 27 gestures (outlier detection, feature selection, data augmentation).

**Figure A2:**
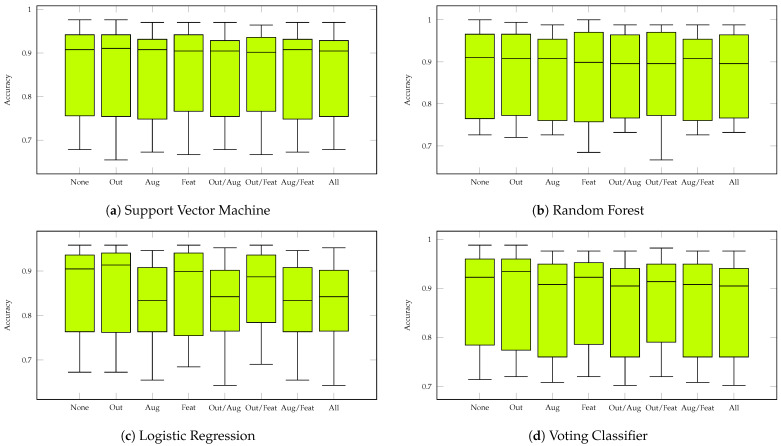
Accuracy values in leave-one-out cross-validation for classifiers with 56 gestures (outlier detection, feature selection, data augmentation).

**Figure A3:**
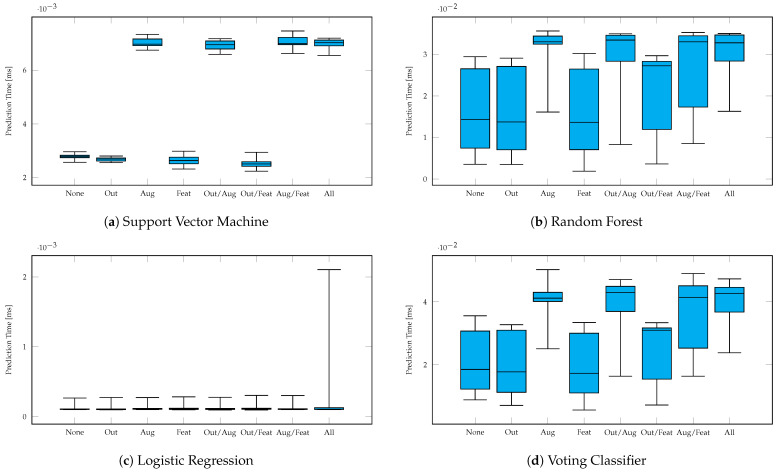
Classification times in leave-one-out cross-validation for classifiers with 27 gestures (outlier detection, feature selection, data augmentation).

**Figure A4:**
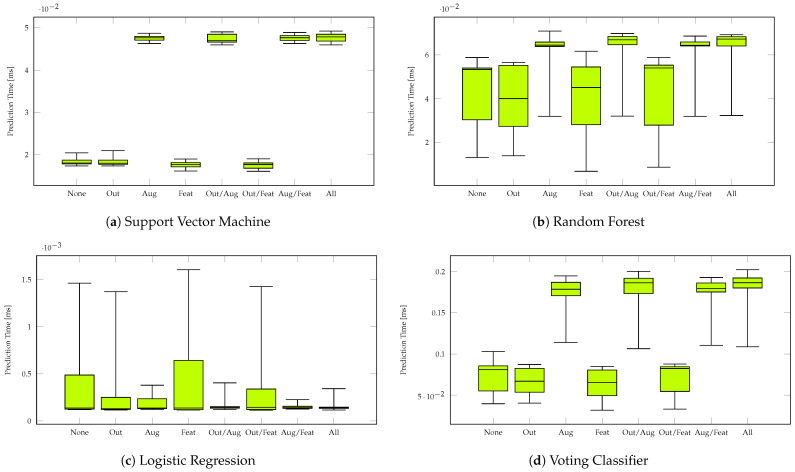
Classification times in leave-one-out cross-validation for classifiers with 56 gestures (outlier detection, feature selection, data augmentation).

Figure A1, Figure A2, Figure A3 and Figure A4 show the plotted metrics of the classifiers with different data preprocessing steps. The black lines mark the range where the metrics of each run can be found (maximum, mean, and minimum). The colored boxes represent the values of the first through third quartiles. Thus, inside a box there are 50% of the determined values from each of the 20 runs.

Independent of the data preprocessing methods used, 27 hand shapes can be classified with an accuracy of 89.14% to 91.91% and 56 hand shapes score 82.86% to 87.50%. In both cases, LR performs worst on average. For 27 hand shapes, VL2 can achieve the highest average accuracy values; for 56 hand shapes, RF performs best. Regardless of the number of hand shapes, there are only 0.51 to 2.53 percentage points between the best and worst feature combinations for each classifier, with the range varying significantly more for 56 hand shapes.

The classification time is the time difference immediately before and after calling the classifiers’ *predict* (https://scikit-learn.org/stable/developers/develop.html (last visited 28 September 2023)) function. It includes the classification of an entire user data set, i.e., up to 168 samples (up to 56 hand shapes with three repetitions) before *outlier detection*. In the case of the VL2 classifier, the classification times of the first layer classifiers are included.

Table 5 and Table 6 show the classification times of all classifiers with respect to the data preprocessing methods used. Again, the best results are marked in green; the worst results are marked in red.

In contrast to the accuracy values, the classification times vary considerably: LR is by far the fastest classifier, with times below 0.3 ms, whereas VL2 understandably takes the longest, with 18.20–40.03 ms for 27 hand shapes and 62.97–179.15 ms for 56 hand shapes, as it contains its own classification in addition to the three other classifiers. It is obvious that the classification times also increase sharply with the number of hand shapes. This affects LR the least (mean two-fold increase in classification time) and SVM the most (mean eight-fold increase in classification time) for the difference from 27 to 56 hand shapes. When doubling the data using *data augmentation*, the times also roughly double.

### 6.1. Machine Learning Classifier

We will now briefly look at the results of the individual ML classifiers before taking a closer look at the data preprocessing methods.

**Support vector machine (SVM)**  showed a robust performance in classifying both data sets. For 27 hand shapes, SVM achieved an average accuracy of 90.46%, while for the more extensive data set with 56 hand shapes, the accuracy dropped slightly to 85.46%. These results suggest a marginal decline in SVM’s efficacy with increasing data complexity.
  In terms of classification time, SVM took between 2.51 ms and 7.06 ms to classify the smaller data set and 17.45–47.76 ms to classify the larger one, indicating good scalability.
**Random forest (RF)**  offers comparable accuracy to SVM, with an average of 90.52% for 27 hand shapes and 86.79% for 56 hand shapes. However, the longer classification times (14.44–30.93 ms for 27 hand shapes and 38.52–62.16 ms for 56 hand shapes) could be a disadvantage in practical applications.
**Logistic regression (LR)**  showed slightly lower accuracy, especially for the larger data set (average 89.58% for 27 hand shapes vs. 84.19% for 56 hand shapes). It can be seen that LR suffers a significant loss of accuracy (2.5–3%) when *data augmentation* is applied to a larger data set. When looking at the learning curves in Figure 10c, it can also be seen that the accuracy decreases as the number of samples increases. It therefore appears that LR has problems with scalability.
  Classification times were the shortest among all classifiers tested, which could make LR an attractive choice for very time-constrained applications, as long as the amount of data is not too high. Regardless of the number of classes, the classification times are below 0.35 ms, but are also the most dependent on processor runtime fluctuations due to these short runtimes. This can also be well-recognized in Figure A3c and Figure A4c. Therefore, comparisons of the classification time for LR should be treated with caution.
**Voting meta-classifier (VL2)**  consistently achieved the highest average accuracy in both data sets (91.50% for 27 hand shapes and 86.59% for 56 hand shapes), if the results for procedures with *data augmentation* in the larger data set were omitted. It seems that the poor scalability of LR affects the accuracy of VL2.
  Classification times were also the longest (18.20–40.03 ms for 27 hand shapes and 62.97–179.15 ms for 56 hand shapes), which may limit its practical applicability in time-critical environments, because it contains all other classifiers in the first layer and additionally its own meta-classification takes place in the second layer.

**Figure 7:**
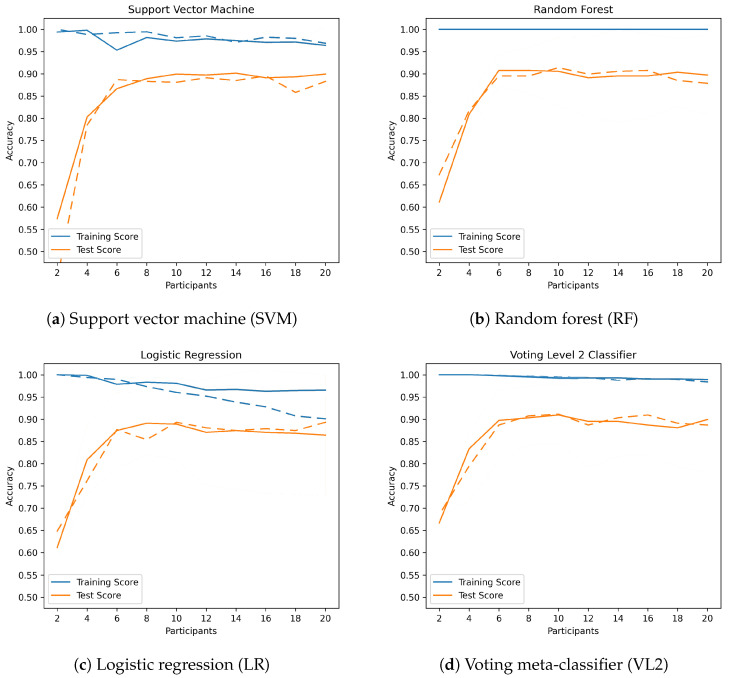
Learning curves with (dashed line) and without (solid line) data augmentation for 27 hand shapes.

### 6.2. Data Preprocessing Methods

Considering the results with respect to the selected data preprocessing methods, it can be said that, with few exceptions, the highest accuracy values are achieved without data preprocessing (except scaling). Occasionally, some combinations of data preprocessing steps and classifiers (e.g., LR with *feature selection* and *data augmentation* for 56 hand shapes) can achieve higher accuracy values, but since the differences are minimal and no real pattern can be recognized, these exceptions are probably due to the choice of hyperparameters. Only VL2 with *outlier detection* shows better accuracy for both 27 and 56 hand shapes compared to VL2 without data preprocessing.

According to Figure A2, the data preprocessing methods for 56 hand shapes and SVM almost all have the same mean, whereas the greatest fluctuations occur for LR. There, the approaches with *data augmentation* are significantly worse than without. For 27 hand shapes (see Figure A1), the fluctuations are lower for all classifiers.

We tested 64 different configurations of data preprocessing (see Table 3 and Table 4). Eight configurations used no data preprocessing, while fifty-six used a combination of the methods described so far. Our tests have shown that only seven of these combinations perform better in terms of accuracy than the runs without data preprocessing.

*Feature selection*, *outlier detection*, and the combination of both lead to improvements in classification times in most cases. The times for LR are difficult to evaluate here, as they are very low and are therefore strongly influenced by runtime fluctuations. *Data augmentation* roughly doubles the classification time when doubling the data.

#### 6.2.1. Outlier Detection

Regarding the *outlier detection*, we found that detecting only very few outliers yielded the best results. Consequently, we set the the maximal distance one point is allowed to have to the closest point within the cluster to one standard deviation per feature on average. With 20 features, this resulted in an *eps* value of 4.4. The *minPoints* parameter was set to 51, as there were a total of 57 samples for each gesture in the training set and we figured that at least 90% of the data should be inliers. These parameters resulted in an average of 3.25 out 1539 samples being considered outliers in the 27-gesture data set. For 56 gestures, an average of 5.8 outliers were found in 3192 samples.

**Figure 8:**
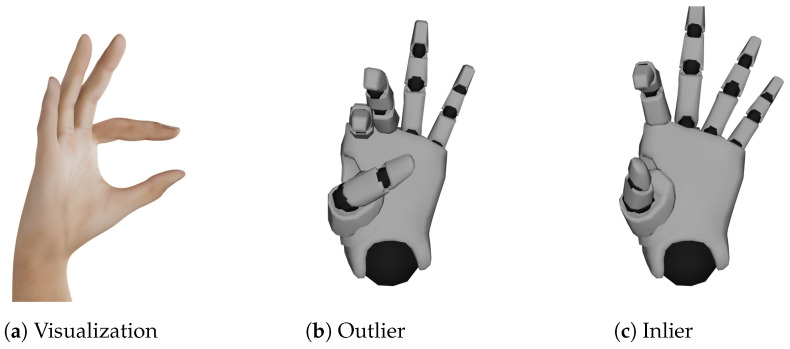
Outlier (bent thumb and middle finger) found for hand shape *Open F* alongside inlier of the same hand shape and visualization.

One example of an outlier alongside an inlier and the visualization of the gesture *Open F* can be seen in Figure 8. The outlier was created by bending the thumb and middle finger too much.

About 0.78% of all samples were identified as outliers, which slightly improved the performance in three of eight cases, as shown in Table 3 and Table 4. More importantly, the classification time improved in six out of eight cases, as seen in Table 5 and Table 6. This leads to a recommendation to use *outlier detection* in time-critical applications.

#### 6.2.2. Data Augmentation

**Figure 9:**
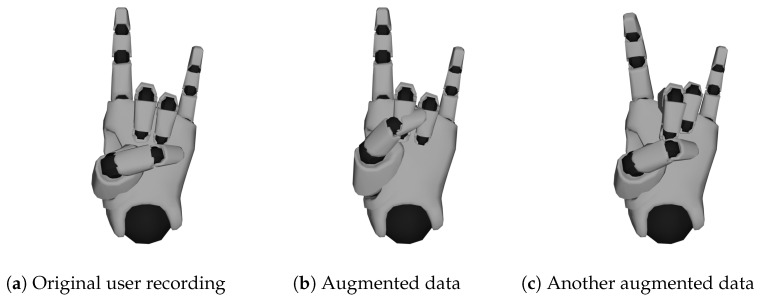
Visualized effect of data augmentation for hand shape *Horns*.

The effect of the applied *data augmentation* method can best be seen in Figure 9, where the result is visualized. Here, two synthetically generated hand shapes for the *Horns* label are shown as an example, along with an original sample for comparison. As can be seen, new data samples can be successfully generated and show some variations in the way the hand shape is performed. Overall, we doubled our training data set using this technique.

**Figure 10:**
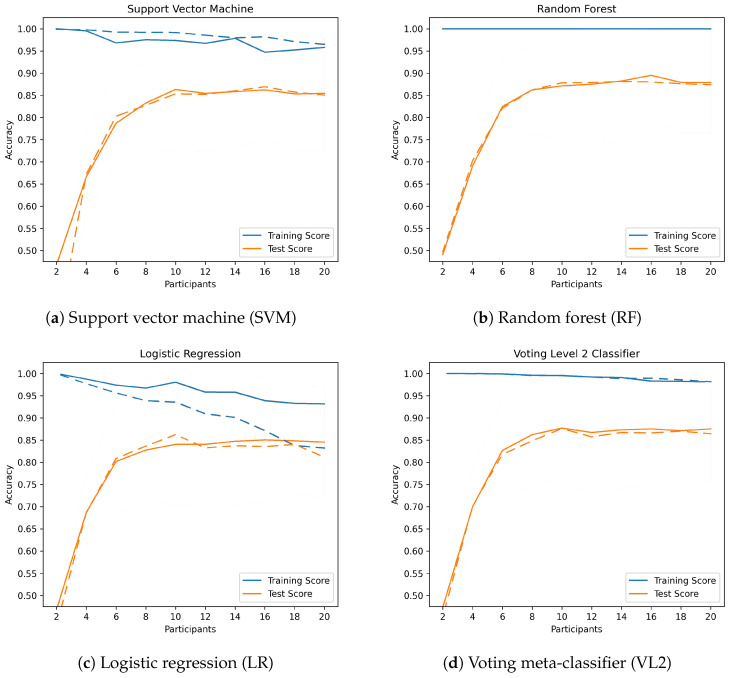
Learning curves with (dashed line) and without (solid line) data augmentation for 56 hand shapes.

As can seen in Table 3 and Table 4, the experimental application of *data augmentation* failed to improve the accuracy further, especially with the larger data set. The reason could be that the collected data are sufficient for the chosen classifiers and, therefore, do not further improve the classification accuracy. As the number of data samples increases, the classification time also increases. To evaluate whether better generalizability can be achieved with *data augmentation*, we created and compared learning curves: We plotted the accuracy obtained without *data augmentation* as a function of the number of participants, i.e., the number and variety of available training data, and compared it with the results when *data augmentation* is applied. Figure 7 and Figure 10 show these learning curves. When the data are not augmented, the curves already show a good fit, with the accuracy on the test set increasing steadily with the number of participants. The *generalization gap*, i.e., the gap between the two curves, is also clearly visible. Just LR shows a significant decrease in training accuracy as the number of data increases (whether due to a larger data set or the use of *data augmention*).

The generalizability can therefore not be increased by augmenting the data, as they already have a high generalizability, with the exception of LR.

Overall, we were able to successfully generate valid synthetic data samples to enrich our training set. For the reasons stated above, the application of the *data augmentation* method is altogether not worth applying in this context and for this type of data. As mentioned in Section 3.2, research on *data augmentation* for wearable sensors has mainly been studied in a dynamic context, where models trained with this more complex type of data benefit more from artificial augmentation of the data set. The application of *data augmentation* would, therefore, be more effective for dynamic gestures and would probably achieve a better effect on accuracy in the area of *deep learning*, as these methods perform significantly better with a large amount of data than the traditional ML methods [34].

#### 6.2.3. Feature Selection

*Feature selection* was used for data preprocessing in a total of four configurations. When applied to both data sets with 20 runs per experiment, we obtained 160 executions. The amount of times each feature was discarded by the algorithm can be seen in Table 7. There were no features discarded in 50 out of 160 runs. The maximum number of discarded features was six (five times in 160 runs). On average, 2.069 features were discarded per run.

According to Table 7, the thumb, index finger, and middle finger seem to be the most important for classification. For example, no thumb stretch features were discarded at all, but the thumb spread feature was discarded more than every fourth time. Features of the index finger were discarded the least, and when they were, only the values of the upper extremities (DIP and PIP stretch features). For the middle finger, each of the four features was discarded at least three times, but the total number of discarded features is lower than for the thumb. The ring and little finger seem to contribute the least amount of information needed to classify gestures, as their features were discarded most often.

It is important to note that the stretch metacarpophalangeal (MCP) value was always preserved for almost all fingers, while the PIP and DIP values were frequently discarded. In most cases, only one of the latter two joints was discarded, while the other was kept for classification. After investigation this phenomenon further, we noticed that these two joints are rarely moved individually. For most gestures, both joints are flexed to about the same degree. Looking at our data, we also noticed that the value of these two joints is often exactly the same, explaining why one of these two joints for each finger was discarded by our feature selection so often. Anatomically, it is not possible to move the upper phalanx (= DIP) independently of the middle phalanx (= PIP) without external influence [9]. This dependence explains why so many values are filtered out here.

Another noteworthy observation is that the ring finger spread value was the most frequently discarded feature and was discarded in more than 40% of all runs. This is likely due to the ring finger barely moving along this axis in most gestures. For example, when spreading your fingers, the ring finger barely moves, while the spread value of all other fingers changes significantly. As the ring finger mainly has a supporting function, its mobility is limited compared to the other fingers. In most cases, the ring finger is used together with its neighboring fingers and therefore exhibits a strong dependence on them. This dependency was obviously recognized by our *feature selection*.

Overall, it can be said that *feature selection* was able to achieve an improvement in classification time with a slight decrease in accuracy. On the other hand, we were able to prove that *feature selection* comprehensibly identified and filtered dependent values. The approach would probably bring even more time advantages if more than 20 features were used for classification.

## 7. Discussion

In this study, we evaluated the efficacy of various ML classifiers and data preprocessing techniques in recognizing hand shapes of ASL. Our focus was not only on achieving high accuracy but also on ensuring real-time applicability. The choice of the most suitable classifier and preprocessing method requires a careful consideration of both accuracy and classification time. The training time is not relevant for our purposes, since the training is performed offline and is not time-critical.

### 7.1. Key Findings

**Accuracy vs. Classification Time Trade-off:**  VL2 achieved the highest accuracy, but was also the slowest, making its use in real-time applications a careful consideration. In contrast, LR offered the best speed but lowest accuracy (2–2.5 percentage points less than VL2). RF and SVM are somewhere in between.
**Impact of Data Preprocessing:**  Data preprocessing techniques such as *feature selection* and *outlier detection* improved the efficiency of classifiers in terms of classification time, but often at the cost of a slight decrease in accuracy. The particular benefit of *data augmentation* could not be proven; instead, it provided poorer accuracy values and higher classification times.

### 7.2. Optimal Classifier for Real-Time Application

In general, the accuracy values achieved are at a comparable level for all classifiers. Since VL2 can almost exclusively achieve the highest accuracy values by combining the advantages of the other classifiers, it is very suitable for our purpose. The high classification time is slightly relativized when considering that in a real-time scenario usually only one hand shape has to be recognized at a time. In our case, the classification time was given for the classification of 82 and 168 hand shapes, respectively, before applying *outlier detection*.

VL2 achieves an average classification time of 20.80 ms for 27 hand shapes and 68.46 ms for 56 hand shapes for data preprocessing without *data augmentation*. If we assume an approximately proportional ratio of classification time to the number of data to be classified, this results in a classification time of approximately 0.25 ms or 0.41 ms for a single hand shape to be classified. Theoretically, classification rates of over 2.45 kHz would be possible, i.e., far more than the 90 Hz sampling rate supported by the data glove used in this study. It can therefore be assumed that a high classification rate for single hand shapes can be achieved even when using hardware that does not perform as well as what we had available.

Regarding data preprocessing, the use of *outlier detection* is recommended, as this leads to improvements, especially in classification times and, when used with VL2, in accuracy. *Feature selection* brought slight improvements in classification time, but these advantages do not add up to those of *outlier detection*, which is why it does not necessarily make sense to use both methods at the same time. The experimental *data augmentation* approach showed no improvements.

Overall, we therefore consider the use of VL2 in conjunction with *outlier detection* to be the most useful for our purpose.

### 7.3. Limitations of Classification

Looking at the confusions within the classification (see Table 8 and Table 9), the types of hand shapes for which there are difficulties in classification can be seen. The data acquisition was supervised, i.e., whether the hand shapes were correctly executed was monitored during the recording. The listed errors are therefore mainly due to the data gloves or the classification methods.

**Figure A5:**
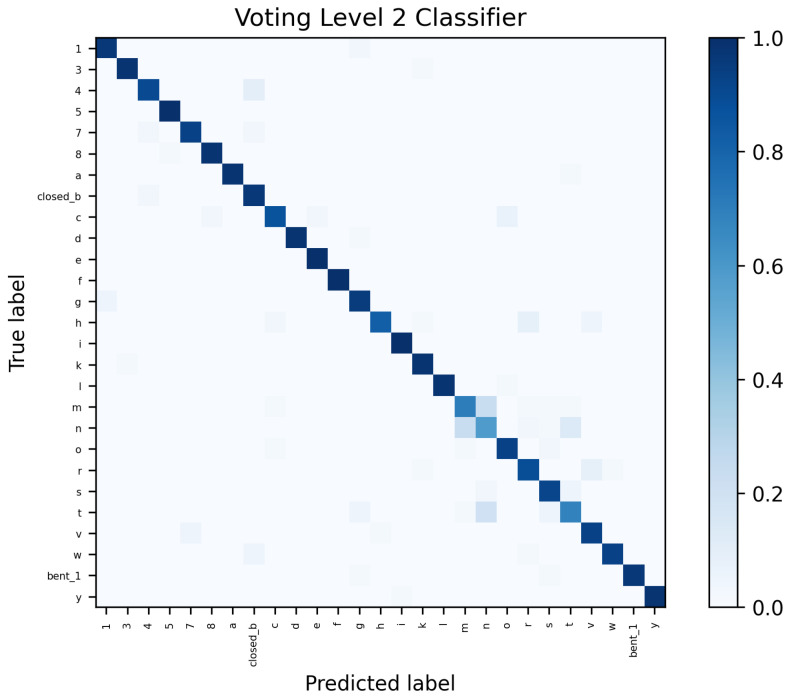
Leave-one-out cross-validation confusion matrix for 27 hand shapes and VL2 classifier with outlier detection.

**Figure A6:**
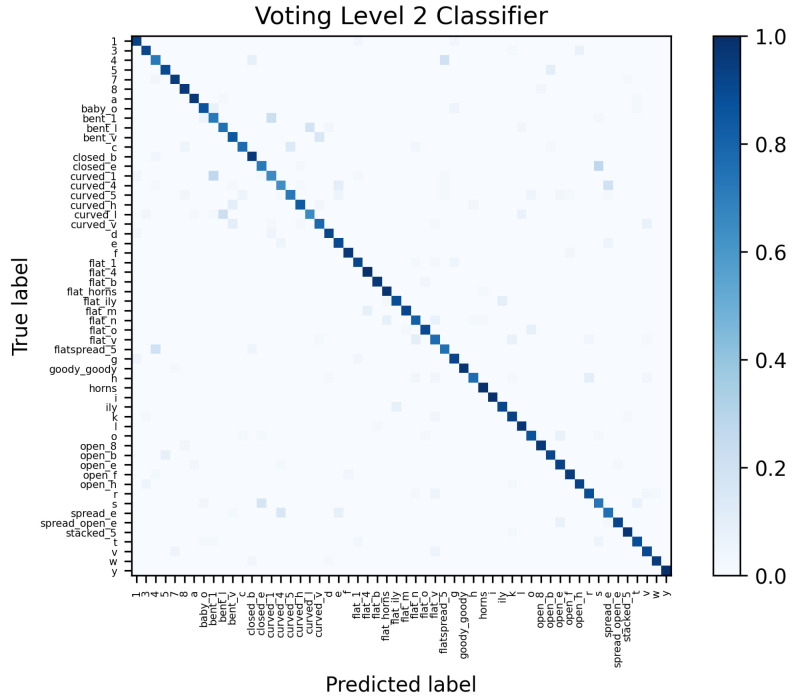
Leave-one-out cross-validation confusion matrix for 56 hand shapes and VL2 classifier with outlier detection.

The corresponding confusion matrices can be viewed in the Appendix A, Figure A5 and Figure A6.

**Figure 11:**
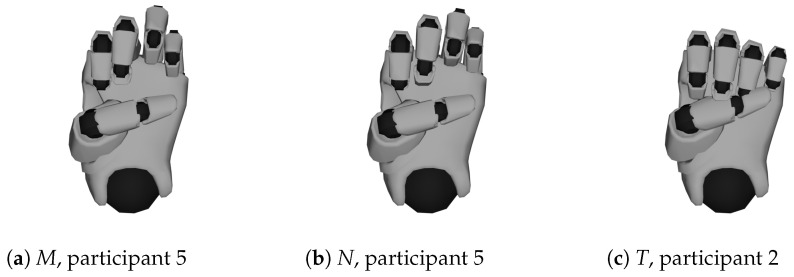
Faulty recordings of hand shapes *M*, *N*, and *T* of participants 2 and 5.

**Thumb Position:**  There are particular difficulties with the hand shapes *M*, *N*, and *T*, where a fist is formed and the thumb crosses a certain number of fingers below. Looking beyond the top 10, it can be seen that *S* is also often interchanged with the hand shapes just mentioned, because here the hand also forms a fist, but the thumb crosses the fingers at the top (and not at the bottom). Moreover, *S* is confused with *Closed E*, where the thumb does not rest on the fingers but directly below them.
  Upon closer inspection of the visualized data, it is noticeable that the position of the thumb is not recorded accurately enough by the data glove (examples can be seen in Figure 11). This is generally a weakness of this glove and seems to be the case with other IMU-controlled gloves [11]. Similarly, it is difficult for the glove to tell whether the thumb is on top of or underneath the crossed fingers.
  The hand shapes *Flat Spread 5* and *4* are also confused and differ only in the position of the thumb.
**Spread Values:**  Another example where the classifiers had difficulties with recognition are the hand shapes *R*, *H*, and *V*, already shown in Figure 3, which differ only in the spread of the index finger and ring finger. The same applies to *4* and *Closed B*.
**Stretch Values:**  The classifiers also often had problems with stretch values, for example, to distinguish between curved and bent hand shapes. Even though the recording of the hand shapes was monitored, it cannot be completely ruled out that the hand shapes were all recorded uniformly, as the difference between bent and curved is sometimes marginal. Examples are *Curved L*⇔*Bent L* and *Curved 1*⇔*Bent 1*. The differences between the hand shapes *Curved 4*⇔*Spread E* and *C*⇔*O* are more significant, but there are also cases of confusion.

Based on our results and the visualizations shown (see Figure 11), we were able to clarify that the limiting factor in the recognition of static hand shapes at this level of accuracy is the hardware used and not the classifiers. To achieve better results, an alternative, more accurate hardware should therefore be considered.

### 7.4. Comparison to Related Work

Pan et al. [9] published a state-of-the-art paper on data gloves in 2023. They examined over 100 English-language papers from reputable publishers and created a comprehensive review that we use for comparison:

**Number of gestures:**  One of their results shows that the papers validate at least three to a maximum of thirty-one hand gestures. The average for static gestures is 20 gestures. Therefore, in comparison, our paper is in the upper range or well above, with 27 and 56 static hand gestures, respectively.
**Number of participants:**  For the number of participants and data recorded (samples), our study is right on the average of 20 participants and 1000 to 10,000 samples (it has 1620 and 3360 samples, and double that if the data are augmented).
**Number of classifiers:**  Most papers examined between three and five classifiers; again, we are in the mean range with four classifiers examined. However, we examine eight different combinations of data preprocessing methods for each classifier.

Table 10 shows us an overview of the related studies [9,11,13,14]. Since most papers ([10,11,12,14]) report their results in a user-dependent manner, we conducted an additional experiment for this purpose. This means that a user’s data can appear in both the training and the test data. For better comparability, we therefore conducted another experiment for both data sets.

This time, randomly combined training and test data were examined in a ratio of 80 to 20. For this, we used VL2 with the already-used hyperparameter ranges and with *outlier detection*, as this was the most promising approach, and trained and tested them 100 times. The training and testing data were randomized again before each run.

An accuracy of 95.55% was achieved for the data set with 27 hand shapes, and 93.19% for 56 hand shapes. Looking at the other classifiers, we see that they are at a similarly high level, between 94.17% (LR) and 95.34% (RF) for 27 hand shapes and between 90.16% (LR) and 93.28% (RF) for 56 hand shapes. It should be noted here that RF can even achieve a slightly higher result than VL2 and that LR obviously scales poorly.

Comparable works, such as Pezzuoli et al.’s [12], achieve an accuracy of up to 99.70% for 27 dynamic gestures, but also use five times as many features. These 96 features include information about hand orientation and movement.

Plawiak et al. [10] use ten sensor values per frame to detect dynamic hand gestures, two of which are rejected using principal component analysis (PCA). They interpolate the average of 60 frames of data to 20 frames, resulting in 160 data points, which they use to classify the 22 different hand gestures. While this gives them a higher accuracy of 98.32% than us (95.92% for 27 hand shapes), they also use eight times the amount of features for classification.

Achenbach et al. [11] achieve a higher accuracy than we do with a comparable number of gestures (25 compared to 27) and features (19 compared to 20) with 99.50%, but they can also rely on information about the orientation of the hands, which we lack.

In direct comparison with the related studies shown in Table 10, we perform slightly worse with 27 hand shapes in terms of accuracy, but we can also rely on significantly less information, which generally has a positive effect on the classification times. With 56 hand shapes, we can distinguish more than twice as many hand shapes as the related studies and this still with a high accuracy.

## 8. Conclusions

In this study, the effects of different data preprocessing steps on the classification of 27 and 56 static hand shapes were investigated. The metrics considered were accuracy and classification time.

According to our research, we can recommend the VL2 classifier with *outlier aetection*, as it has a high accuracy with an acceptable classification time. With this setting, 91.91% (27 hand shapes) and 87.50% (56 hand shapes) accuracy could be achieved for user-independent tests, with a classification time of less than 15 ms and less than 60 ms, respectively. In user-dependent tests, as much as 95.55% and 93.28% accuracy could be achieved. Comparable studies with better accuracy values either had more information available to classify the data or were only able to distinguish significantly fewer classes. For very time-critical applications, LR with *outlier detection* can also be used, whose accuracy is slightly lower, but classification time is significantly higher. This recommendation is independent of the number of hand shapes to be classified.

The use of *feature selection* brought a slight improvement in classification times, but this is not necessarily additive to the advantages of *outlier detecion*. With more features, the advantages of *feature selection* would certainly be greater. Our approach to *data augmentation* was able to double the number of training data with valid data; however, no improvement in accuracy or generalizability was observed. This is certainly due to the fact that we classified static gestures, whereas related studies have pointed to noticeable improvements for dynamic gestures.

### Outlook

Future work will evaluate how the data preprocessing methods would behave with dynamic data with significantly more features. Especially improvements in *data augmentation* and *feature selection* could then be expected. *Data augmentation* should lead to higher accuracy and better generalizability, whereas *feature selection* should lead to faster classification times.

In this paper, we have examined and compared some traditional classifiers in detail, so it would be exciting to know how *deep-learning* algorithms would perform in comparison. The development and optimization of these classifiers could be one of the main topics of the next study.

It would also be conceivable to include hand orientation in the future. This would increase the number of features from the current 20 to 24. This additional information could help to further increase accuracy and classify an even greater number of different hand shapes. It would then also be possible to classify not only hand shapes, but simple signs, such as letters from the finger alphabet, as well. A distinction between, for example, *K* and *P*, which share the same hand shape (see Section 1), would then be possible.

We hope to achieve a further significant improvement in accuracy by using a new generation of data gloves. The *Manus Quantum Precision XR* data gloves use magnetic tracking instead of an IMU and flex sensor-based tracking. With this, the position of the various sensors in the glove is measured by detecting fluctuations in the magnetic field around the glove [35]. Especially the problems with the thumb position (see Figure 11) could be solved with this new technology. Overall, the new glove makes a very accurate impression in initial tests.

## Figures and Tables

**Table 1:** Features and joint values with their respective range of motion (normalized output of Manus Core SDK and corresponding degree range). Names of joints can be seen in Figure 6.

Feature	Finger	Joint Value	SDK Range		Degree Range	
			Min	Max	Min	Max
0	Thumb	Spread CMC	0.0	1.0	$5^{\circ }$	$50^{\circ }$
1	Index	Spread MCP	−1.0	1.0	$-20^{\circ }$	$20^{\circ }$
2	Middle	Spread MCP	−1.0	1.0	$-20^{\circ }$	$20^{\circ }$
3	Ring	Spread MCP	−1.0	1.0	$-20^{\circ }$	$20^{\circ }$
4	Pinky	Spread MCP	−1.0	1.0	$-20^{\circ }$	$20^{\circ }$
5	Thumb	Stretch CMC	0.0	1.0	$-20^{\circ }$	$25^{\circ }$
6	Thumb	Stretch MCP	0.0	1.0	$-20^{\circ }$	$45^{\circ }$
7	Thumb	Stretch IP	0.0	1.0	$-15^{\circ }$	$80^{\circ }$
8	Index	Stretch MCP	0.0	1.0	$0^{\circ }$	$80^{\circ }$
9	Index	Stretch PIP	0.0	1.0	$0^{\circ }$	$100^{\circ }$
10	Index	Stretch DIP	0.0	1.0	$0^{\circ }$	$90^{\circ }$
11	Middle	Stretch MCP	0.0	1.0	$0^{\circ }$	$80^{\circ }$
12	Middle	Stretch PIP	0.0	1.0	$0^{\circ }$	$100^{\circ }$
13	Middle	Stretch DIP	0.0	1.0	$0^{\circ }$	$90^{\circ }$
14	Ring	Stretch MCP	0.0	1.0	$0^{\circ }$	$80^{\circ }$
15	Ring	Stretch PIP	0.0	1.0	$0^{\circ }$	$100^{\circ }$
16	Ring	Stretch DIP	0.0	1.0	$0^{\circ }$	$90^{\circ }$
17	Pinky	Stretch MCP	0.0	1.0	$0^{\circ }$	$80^{\circ }$
18	Pinky	Stretch PIP	0.0	1.0	$0^{\circ }$	$100^{\circ }$
19	Pinky	Stretch DIP	0.0	1.0	$0^{\circ }$	$90^{\circ }$

**Table 2:** Hyperparameter optimization ranges for our experiments.

Classifier	Parameter	Pre-Grid Search Range	Grid Search Range
**SVM**	*C*	$2^{-5},2^{-3},\ldots ,2^{15}$	$2^{0},2^{1},\ldots ,2^{4}$
	γ	$2^{-15},2^{-14},\ldots ,2^{5}$	$2^{-8},2^{-7},\ldots ,2^{-2}$
**RF**	*criterion*	gini, entropy	gini, entropy
	*max_features*	1,2,…,10	1,2,…,6
	*n_estimators*	$2^{0},2^{1},\ldots ,2^{10}$	$2^{6},2^{7},\ldots ,2^{10}$
**LR**	*penalty*	elasticnet	elasticnet
	*solver*	newton-cg, lbfgs, sag, saga	saga
	*C*	$2^{-5},2^{-3},\ldots ,2^{15}$	$2^{-5},2^{-4},\ldots ,2^{4}$
	*l_1__ratio*	0.1,0.2,…,1	0.2,0.3,…,0.5
	*penalty*	none, l_1_, l_2_	$l_{2}$
	*solver*	newton-cg, lbfgs, sag, saga	newton-cg, lbfgs, sag, saga
	*C*	$2^{-5},2^{-3},\ldots ,2^{15}$	$2^{-5},2^{-4},\ldots ,2^{4}$

**Table 3:** Mean accuracy values of leave-one-out cross-validation in dependence of different data preprocessing methods for 27 hand shapes (outlier detection, data augmentation, feature selection).

Data Preprocessing			Machine Learning Classifier				Results		
Out	Aug	Feat	SVM	RF	LR	VL2	Mean	Min	Max
✗	✗	✗	0.9080	0.9123	0.8963	0.9160	0.9082	0.8963	0.9160
✗	✗	✓	0.9037	0.9105	0.8951	0.9185	0.9069	0.8951	0.9185
✗	✓	✗	0.9037	0.9037	0.8914	0.9123	0.9028	0.8914	0.9123
✗	✓	✓	0.9031	0.9049	0.9000	0.9154	0.9059	0.9000	0.9154
✓	✗	✗	0.9080	0.9043	0.8981	0.9191	0.9074	0.8981	0.9191
✓	✗	✓	0.9043	0.9037	0.8981	0.9111	0.9043	0.8981	0.9111
✓	✓	✗	0.9031	0.9025	0.8951	0.9142	0.9037	0.8951	0.9142
✓	✓	✓	0.9025	0.9000	0.8920	0.9130	0.9019	0.8920	0.9130
Mean			0.9046	0.9052	0.8958	0.9150			
Min			0.9025	0.9000	0.8914	0.9111			
Max			0.9080	0.9052	0.9000	0.9191			

**Table 4:** Mean accuracy values of leave-one-out cross-validation in dependence of different data preprocessing methods for 56 hand shapes (outlier detection, data augmentation, feature selection).

Preprocessing			Machine Learning Classifier				Results		
Out	Aug	Feat	SVM	RF	LR	VL2	Mean	Min	Max
✗	✗	✗	0.8610	0.8714	0.8542	0.8744	0.8653	0.8542	0.8744
✗	✗	✓	0.8571	0.8661	0.8563	0.8711	0.8626	0.8563	0.8711
✗	✓	✗	0.8515	0.8664	0.8298	0.8598	0.8519	0.8298	0.8598
✗	✓	✓	0.8515	0.8664	0.8298	0.8598	0.8519	0.8298	0.8598
✓	✗	✗	0.8568	0.8696	0.8539	0.8750	0.8638	0.8539	0.8750
✓	✗	✓	0.8554	0.8646	0.8539	0.8711	0.8612	0.8539	0.8711
✓	✓	✗	0.8518	0.8693	0.8286	0.8580	0.8519	0.8286	0.8580
✓	✓	✓	0.8518	0.8693	0.8286	0.8580	0.8519	0.8286	0.8580
Mean			0.8546	0.8679	0.8419	0.8659			
Min			0.8515	0.8646	0.8286	0.8580			
Max			0.8568	0.8696	0.8539	0.8750			

**Table 5:** Mean classification times of leave-one-out cross-validation in dependence of different data preprocessing methods for 27 hand shapes (outlier detection, feature selection, data augmentation).

Data Preprocessing			Machine Learning Classifier				Results		
Out	Aug	Feat	SVM	RF	LR	VL2	Mean	Min	Max
✗	✗	✗	2.780	16.579	0.112	21.099	10.143	0.112	21.099
✗	✗	✓	2.635	14.444	0.126	18.198	8.851	0.126	18.198
✗	✓	✗	7.026	30.926	0.124	40.029	19.526	0.124	40.029
✗	✓	✓	7.059	26.052	0.117	35.778	17.252	0.117	35.778
✓	✗	✗	2.677	16.323	0.127	20.256	9.846	0.127	20.256
✓	✗	✓	2.506	20.138	0.120	23.664	11.607	0.120	23.664
✓	✓	✗	6.931	28.818	0.117	38.409	18.569	0.117	38.409
✓	✓	✓	6.991	29.491	0.224	39.082	18.947	0.224	39.082
Mean			4.826	22.846	0.133	29.564			
Min			2.506	14.444	0.117	18.198			
Max			7.059	30.926	0.224	40.029			

**Table 6:** Mean classification times of leave-one-out cross-validation in dependence of different data preprocessing methods for 56 hand shapes (outlier detection, feature selection, data augmentation).

Data Preprocessing			Machine Learning Classifier				Results		
Out	Aug	Feat	SVM	RF	LR	VL2	Mean	Min	Max
✗	✗	✗	18.245	44.271	0.365	72.972	33.963	0.365	72.972
✗	✗	✓	17.594	38.516	0.441	62.973	29.881	0.441	62.973
✗	✓	✗	47.519	62.163	0.186	173.830	70.924	0.186	173.830
✗	✓	✓	47.601	62.045	0.149	174.654	71.112	0.149	174.654
✓	✗	✗	18.254	40.345	0.335	67.490	31.606	0.335	67.490
✓	✗	✓	17.486	42.682	0.338	70.423	32.732	0.338	70.423
✓	✓	✗	47.473	63.251	0.170	177.552	72.111	0.170	177.552
✓	✓	✓	47.760	63.055	0.164	179.149	72.532	0.164	179.149
Mean			32.741	52.041	0.268	122.380			
Min			17.486	38.516	0.149	62.973			
Max			47.760	63.251	0.338	179.149			

**Table 7:** Features and number of times they were discarded by ga.

Feature	Finger	Joint Value	Discarded	Discarded	Discarded
			Absolute	Relative	Total
0	Thumb	Spread CMC	47	29.38%	47
5	Thumb	Stretch CMC	0	-	
6	Thumb	Stretch MCP	0	-	
7	Thumb	Stretch IP	0	-	
1	Index	Spread MCP	0	-	19
8	Index	Stretch MCP	0	-	
9	Index	Stretch PIP	10	6.25%	
10	Index	Stretch DIP	9	5.62%	
2	Middle	Spread MCP	3	1.88%	37
11	Middle	Stretch MCP	3	1.88%	
12	Middle	Stretch PIP	15	9.38%	
13	Middle	Stretch DIP	16	10.00%	
3	Ring	Spread MCP	66	41.25%	129
14	Ring	Stretch MCP	5	3.12%	
15	Ring	Stretch PIP	36	22.50%	
16	Ring	Stretch DIP	22	13.75%	
4	Pinky	Spread MCP	24	15.00%	99
17	Pinky	Stretch MCP	9	5.62%	
18	Pinky	Stretch PIP	30	18.75%	
19	Pinky	Stretch DIP	36	22.50%	

**Table 8:** Top ten most common classification confusions for 27 hand shapes.

True Label	Predicted Label	Confusion Rate
N	M	0.2333
M	N	0.2333
T	N	0.2000
N	T	0.1333
4	Closed B	0.1000
R	V	0.0833
H	R	0.0833
C	O	0.0667
V	7	0.0500
W	Closed B	0.0500

**Table 9:** Top ten most common classification confusions for 56 hand shapes.

True Label	Predicted Label	Confusion Rate
Closed E	S	0.2667
Curved 1	Bent 1	0.2667
Bent 1	Curved 1	0.2167
Curved L	Bent L	0.2167
Curved 4	Spread E	0.2000
Flat Spread 5	4	0.2000
4	Flat Spread 5	0.2000
Bent L	Curved L	0.1833
S	Closed E	0.1667
Spread E	Curved 4	0.1500

**Table 10:** Comparison of user-dependent recognition accuracy with related studies, ordered by number of gestures (NoG) and number of participants.

Author(s)	Classifier	Type	HS	Mo	Or	NoG	NoP	Accuracy
Achenbach et al. [11]	SVM	Hand shapes of rock–paper–scissors	✓			5	30	99.20%
Shukor et al. [13]	Distance	Hand gestures of Malaysian Sign Language	✓	✓		9	4	88.88%
Saggio et al. [14]	CNN	Signs of Italian Sign Language	✓	✓		10	7	98.00%
Plawiak et al. [10]	SVM	Hand–body language gestures, e.g., *Okay* sign	✓	✓	✓	22	10	98.32%
Achenbach et al. [11]	SVM	Hand gestures of rock–paper–scissors	✓		✓	25	9	99.50%
Pezzuoli et al. [12]	SVM	Simple hand gestures, e.g., clockwise rotation	✓	✓	✓	27	5	99.70%
**This study**	**VL2**	**Hand shapes of ASL finger alphabet**	**✓**			**27**	**20**	**95.55%**
**This study**	**RF**	**Hand shapes of ASL-Lex [5]**	**✓**			**56**	**20**	**93.28%**

The following abbreviations are used: hand shape (HS), movement (Mo), orientation (Or), number of gestures (NoG), number of participants (NoP).

## Data Availability

The data we used and the results of this study can be found at https://figshare.com/articles/dataset/64_ASL_Hand_Shapes_Data_Glove_Recordings/24768714. The hand shapes we used can be found at https://github.com/serious-games-darmstadt/sign-visualizations/tree/main/handshapes.

## Abbreviations

The following abbreviations are used in this manuscript:

ACM	Association for Computing Machinery
ASL	American Sign Language
CMC	Carpometacarpal
DBSCAN	Density-Based Spatial Clustering of Applications with Noise
DIP	Distal Interphalangea
DoF	Degrees of Freedom
DT	Decision Tree
GA	Genetic Algorithm
IEEE	Institute of Electrical and Electronics Engineers
IP	Interphalangeal
IMU	Inertial Measurement Unit
LR	Logistic Regression
MCP	Metacarpophalangeal
ML	Machine Learning
OvA	One-versus-Al
OvO	One-versus-One
PCA	Principal Component Analysis
PIP	Proximal Interphalangeal
RF	Random Forest
SVM	Support Vector Machine
VL2	Voting Meta-Classifier
VR	Virtual Reality
WoS	Web of Science

## Appendix A

**Table A1:** Used hand shapes of ASL finger alphabet (*1* to *Y*) and ASL-Lex (all hand shapes except *M* and *N*).

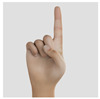
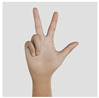
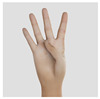
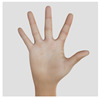
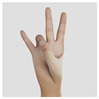
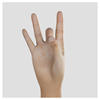
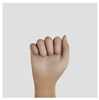

						
1	3	4	5	7	8	A

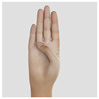
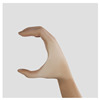
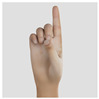
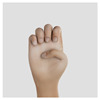
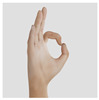
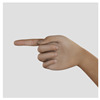
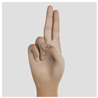
						
Closed B	C	D	E	F	G	H

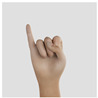
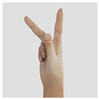
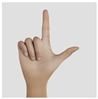
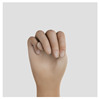
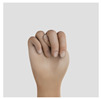
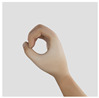
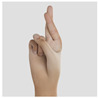
						
I	K	L	M	N	O	R

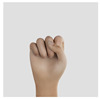
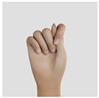
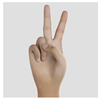
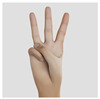
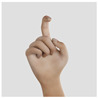
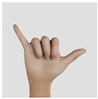
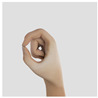
						
S	T	V	W	Bent 1	Y	Baby O

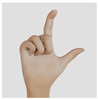
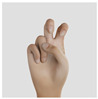
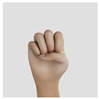
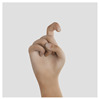
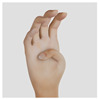
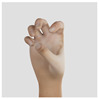
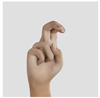
						
Bent L	Bent V	Closed E	Curved 1	Curved 4	Curved 5	Curved H

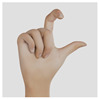
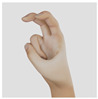
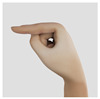
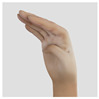
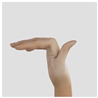
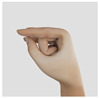
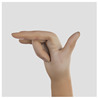
						
Curved L	Curved V	Flat 1	Flat 4	Flat B	Flat Horns	Flat ILY

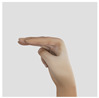
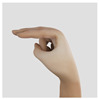
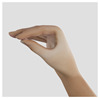
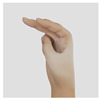
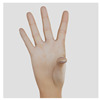
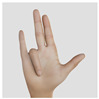
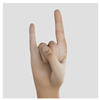
						
Flat M	Flat N	Flat O	Flat V	Flat Spread 5	Goody Goody	Horns

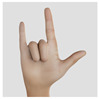
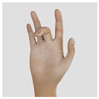
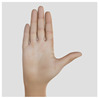
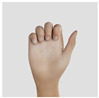
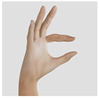
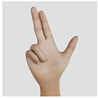
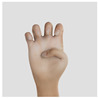
						
ILY	Open 8	Open B	Open E	Open F	Open H	Spread E

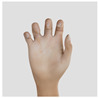
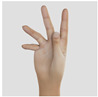
						
Spread Open E	Stacked 5					

Hand shapes can be viewed in detail at https://sign-parametrization.netlify.app/handshapes.

## References

[1] World Health Organization (2021). Deafness and Hearing Loss.

[2] Stokoe W.C., Casterline D.C., Croneberg C.G. (1976). A Dictionary of American Sign Language on Linguistic Principles.

[3] Stokoe W. (1960). Sign Language Structure. 1978.

[4] Achenbach P., Göksu Y., Kullmann T., Tregel T., Göbel S. Towards handshape identification for automatic gesture recognition using sign notation systems. Proceedings of the 8th European Conference on Social Media (ECSM ’21).

[5] Sehyr Z.S., Caselli N., Cohen-Goldberg A.M., Emmorey K. (2021). The ASL-LEX 2.0 Project: A Database of Lexical and Phonological Properties for 2,723 Signs in American Sign Language. J. Deaf Stud. Deaf Educ..

[6] Caselli N.K., Sehyr Z.S., Cohen-Goldberg A.M., Emmorey K. (2017). ASL-LEX: A lexical database of American Sign Language. Behav. Res. Methods.

[7] Brentari D. (1998). A Prosodic Model of Sign Language Phonology.

[8] Fricke E., Bressem J. (2020). Gesten—Gestern, Heute, üBermorgen. Vom Forschungsprojekt zur Ausstellung.

[9] Pan M., Tang Y., Li H. (2023). State-of-the-Art in Data Gloves: A Review of Hardware, Algorithms, and Applications. IEEE Trans. Instrum. Meas..

[10] Plawiak P., Sosnicki T., Niedzwiecki M., Tabor Z., Rzecki K. (2016). Hand Body Language Gesture Recognition Based on Signals from Specialized Glove and Machine Learning Algorithms. IEEE Trans. Ind. Inform..

[11] Achenbach P., Purdack D., Wolf S., Müller P.N., Tregel T., Göbel S., Söbke H., Spangenberger P., Müller P., Göbel S. (2022). Paper Beats Rock: Elaborating the Best Machine Learning Classifier for Hand Gesture Recognition. Serious Games.

[12] Pezzuoli F., Corona D., Corradini M.L. (2021). Recognition and Classification of Dynamic Hand Gestures by a Wearable Data-Glove. SN Comput. Sci..

[13] Shukor A.Z., Miskon M.F., Jamaluddin M.H., Ali@Ibrahim F.b., Asyraf M.F., Bahar M.B.b. (2015). A New Data Glove Approach for Malaysian Sign Language Detection. Procedia Comput. Sci..

[14] Saggio G., Cavallo P., Ricci M., Errico V., Zea J., Benalcázar M.E. (2020). Sign Language Recognition Using Wearable Electronics: Implementing k-Nearest Neighbors with Dynamic Time Warping and Convolutional Neural Network Algorithms. Sensors.

[15] Kunz N. (2022). Recognition and Classification of Handshapes of American Finger Alphabet. Bachelor’s Thesis.

[16] Pedregosa F., Varoquaux G., Gramfort A., Michel V., Thirion B., Grisel O., Blondel M., Prettenhofer P., Weiss R., Dubourg V. (2011). Scikit-learn: Machine Learning in Python. J. Mach. Learn. Res..

[17] Ali S., Smith-Miles K.A. (2006). Improved Support Vector Machine Generalization Using Normalized Input Space. AI 2006: Advances in Artificial Intelligence.

[18] Ghojogh B., Crowley M. (2019). The Theory Behind Overfitting, Cross Validation, Regularization, Bagging, and Boosting: Tutorial. arXiv.

[19] Zhang Y., Zheng Y., Qian K., Zhang G., Liu Y., Wu C., Yang Z. (2021). Widar3.0: Zero-Effort Cross-Domain Gesture Recognition with Wi-Fi. IEEE Trans. Pattern Anal. Mach. Intell..

[20] Palipana S., Salami D., Leiva L.A., Sigg S. (2021). Pantomime: Mid-air gesture recognition with sparse millimeter-wave radar point clouds. Proc. ACM Interact. Mob. Wearable Ubiquitous Technol..

[21] Ohashi H., Al-Naser M., Ahmed S., Akiyama T., Sato T., Nguyen P., Nakamura K., Dengel A. Augmenting Wearable Sensor Data with Physical Constraint for DNN-Based Human-Action Recognition. Proceedings of the Time Series Workshop.

[22] Um T.T., Pfister F.M.J., Pichler D., Endo S., Lang M., Hirche S., Fietzek U., Kulić D. Data augmentation of wearable sensor data for parkinson’s disease monitoring using convolutional neural networks. Proceedings of the 19th ACM International Conference on Multimodal Interaction.

[23] Liu S., Ostadabbas S., Leal-Taixé L., Roth S. (2019). A Semi-supervised Data Augmentation Approach Using 3D Graphical Engines. Computer Vision—ECCV 2018 Workshops.

[24] Blender Online Community (2018). Blender—A 3D Modelling and Rendering Package.

[25] Feix T. (2011). Anthropomorphic Hand Optimization Based on a Latent Space Analysis. Ph.D. Thesis.

[26] Baraniuk R.G., Cevher V., Wakin M.B. (2010). Low-dimensional models for dimensionality reduction and signal recovery: A geometric perspective. Proc. IEEE.

[27] Whitley D. (1994). A genetic algorithm tutorial. Stat. Comput..

[28] Li D.J., Li Y.Y., Li J.X., Fu Y. (2018). Gesture Recognition Based on BP Neural Network Improved by Chaotic Genetic Algorithm. Int. J. Autom. Comput..

[29] Cunningham P., Cord M., Delany S.J. (2008). Supervised Learning. Machine Learning Techniques for Multimedia.

[30] Galar M., Fernández A., Barrenechea E., Bustince H., Herrera F. (2011). An overview of ensemble methods for binary classifiers in multi-class problems: Experimental study on one-vs-one and one-vs-all schemes. Pattern Recognit..

[31] Breiman L. (2001). Random Forests. Mach. Learn..

[32] Srimaneekarn N., Hayter A., Liu W., Tantipoj C. (2022). Binary response analysis using logistic regression in dentistry. Int. J. Dent..

[33] Zappi P., Lombriser C., Stiefmeier T., Farella E., Roggen D., Benini L., Tröster G. (2008). Activity recognition from on-body sensors: Accuracy-power trade-off by dynamic sensor selection. Wireless Sensor Networks, Proceedings of the 5th European Conference, EWSN 2008, Bologna, Italy, 30 January–1 February 2008; Proceedings.

[34] Sarker I.H. (2021). Machine Learning: Algorithms, Real-World Applications and Research Directions. SN Comput. Sci..

[35] Cox C.M.J., Hicks B., Gopsill J., Snider C. (2023). From haptic interaction to design insight: An empirical comparison of commercial hand-tracking technology. Proc. Des. Soc..

